# Risks of illness in the work of the nursing team in a psychiatric hospital^1^

**DOI:** 10.1590/1518-8345.2458.3032

**Published:** 2018-08-09

**Authors:** Kayo Henrique Jardel Feitosa Sousa, Tayane Silva Gonçalves, Marize Barbosa Silva, Elizabeth Camacho Fonseca Soares, Maria Luiza Figueiredo Nogueira, Regina Célia Gollner Zeitoune

**Affiliations:** 2. MSc.; 3 Master’s student, Escola de Enfermagem Anna Nery, Universidade Federal do Rio de Janeiro, Rio de Janeiro, RJ, Brazil. Scholarship holder at Coordenação de Aperfeiçoamento de Pessoal de Nível Superior (CAPES), Brazil.; 4 Master’s student, Escola de Enfermagem Anna Nery, Universidade Federal do Rio de Janeiro, Rio de Janeiro, RJ, Brazil. RN, Hospital Universitário, Universidade Federal do Rio de Janeiro, Rio de Janeiro, RJ, Brazil.; Universidade Federal do Rio de Janeiro, Hospital Universitário, Universidade Federal do Rio de Janeiro, Rio de Janeiro, RJ, Brazil; 5 Master’s student, Escola de Enfermagem Anna Nery, Universidade Federal do Rio de Janeiro, Rio de Janeiro, RJ, Brazil. Professor, Fundação de Apoio à Escola Técnica do Estado do Rio de Janeiro, Rio de Janeiro, RJ, Brazil.; Fundação de Apoio à Escola Técnica do Estado do Rio de Janeiro, Rio de Janeiro, RJ, Brazil; 6 Doctoral student, Escola de Enfermagem Anna Nery, Universidade Federal do Rio de Janeiro, Rio de Janeiro, RJ, Brazil. RN, Instituto Nacional do Câncer, Rio de Janeiro, RJ, Brazil.; Instituto Nacional do Câncer, Instituto Nacional do Câncer, Rio de Janeiro, RJ, Brazil; 7 PhD, Full Professor, Escola de Enfermagem Anna Nery, Universidade Federal do Rio de Janeiro, Rio de Janeiro, RJ, Brazil.

**Keywords:** Nursing, Working Conditions, Occupational Health, Mental Health, Human Resources, Psychiatric Hospitals

## Abstract

**Objective:**

To analyze the risks of illnesses related to the work context of nursing workers in a psychiatric hospital.

**Method:**

Cross-sectional and quantitative study, developed in a psychiatric hospital with 74 nursing workers. The Work Context Assessment Scale was used to measure the risks of illness at work. Descriptive analyzes were performed with mean and standard deviation. To test the reliability of the data, the Cronbach’s alpha test was used. The correlation between the factors of the work context was tested using the Spearman correlation coefficient.

**Results:**

The organization of work was considered serious, social-professional relationships were considered satisfactory and working conditions were considered severe for risks of illness at work. The repetitiveness of tasks, work conditions that pose risks to safety, inadequate furniture and physical structure of the workplace, and the existence of noise in the work environment were indicated as severe risks to workers’ health.

**Conclusion:**

The factor working conditions is the one that contributes the most to illness among nursing workers.

## Introduction

The evaluation of mental health services has been constant in recent years. These investigations, were partially, driven by changes in the care model, which used to be hospital-based and is now based on the community and the territory covered. In this new model of care, the qualification, health, satisfaction and commitment of the worker are directly related to the quality of the services offered^1^ ^-^ ^2^.

The World Health Organization (WHO) recommends evaluating mental health services^3^ and recognizes work as a source of pleasure and sometimes of suffering^4^. However, there is little discussion in national and international literature about the nursing work process, especially in mental health services^5^. Therefore, given the need for new approaches to research on workers health^6^, this study evaluated the nursing work context in a psychiatric hospital aiming to analyze the risk of illness.

Another justification for this study was the fact that, among the professional categories in the health sector, nursing professionals are the ones with the highest rates of occupational accidents and health problems. This is considered a consequence of uninterrupted care, which implies in continuous contact with the patients and their families, resulting in exhaustion and a high workload^7^. In addition, these professionals, and especially psychiatric nursing workers, are exposed to higher workloads and unfavorable working conditions that generate stress, emotional tension and physical and mental exhaustion, which can lead to work-related illnesses^1^. These data are similar to the findings of a study conducted with pharmacists in France, who had the responsibility of performing their tasks efficiently and without mistakes, while exposed to stress factors and bearing the emotional load of patients and families, in addition to being under the pressure of entrepreneurial responsibilities^8^.

At the core of this discussion, it is emphasized that the work context is the social space where social-professional relationships occur, and where the organization of work and working conditions take place^9^. The organization of work includes questions regarding its division, hierarchy, productivity, formal rules, working hours, time for breaks and rest, pace of work, nature of the activity and control over work^10^. The social-professional relationships represent the model of management, communication and professional interaction in the work environment^11^. Working conditions are represented by the environment where work occurs and the way it is developed, all related to the interaction between a set of social, psychic, biological and material elements and circumstances, influenced by economic, organizational and technical factors^12^.

Given these considerations, the objective of this study was to assess the risk of illness related to the work context among nursing workers in a psychiatric hospital.

## Method

This is a cross-sectional and quantitative study, conducted in a psychiatric hospital, that is, a reference center for the care of patients with mental disorders, located in Teresina, Piauí. The study population consisted of 93 nursing workers of both genders, who were in the hospital during the period of data collection. Data were collected between March and April 2016, in a single stage, through an interview. The inclusion criterion was being part of the nursing team, while the exclusion criteria were to be on vacation or leave or being away from the care functions.

Of the 93 eligible nursing workers, 74 (82.2%) participated in the study. Three were on leave. The losses corresponded to eight that refused to participate and eight that were not found during the data collection period.

The results presented refer to the Work Context Assessment Scale (EACT), a sub-scale of the Inventory on Work and Risk of Illness (ITRA), developed in 2003 by Ana Magnólia Mendes and Mário César Ferreira, later validated by Ana Magnólia Mendes and collaborators in the years 2004 and 2006, which is in the public domain^11^. The EACT is a Likert-type scale composed of 31 questions divided into three factors: organization of work (11 questions), social-professional relationships (10 questions) and working conditions (10 questions). Its interpretation is based on questions (items) and factors^11^, and the reliability of the factors is evaluated by estimating the internal consistency using Cronbach’s Alpha Coefficient (α).

The data collected were analyzed in the software Statistical Package for Social Sciences (SPSS) version 21.0, with descriptive analysis of each item and later of each factor, considering arithmetic means and standard deviations (SD). The correlation between EACT factors was analyzed using the Spearman correlation coefficient.

The classification of the risk of illness considered the values ​​established by the authors of the instrument: means above 3.70 - more negative evaluation, severe risk of illness; between 3.69 and 2.30 - moderate evaluation, serious risk of illness; below 2.29 - satisfactory evaluation, with an environment that can favor worker’s health^11^.

The research was approved by the Research Ethics Committe, under protocol number 1,434,109, after acceptance of the place where the data was collected. All study participants read, signed and received an Informed Consent Form.

## Results

The majority of the participants were female (91.9%, n=68), with a mean age of 49 years (±9.22), with no partner (54.1%, n=40), and were black or Asian (65,8%, n=48). Regarding the professional aspects, there were 81.1% (n=60) nursing assistants/technicians and 18.9% (n=14) nurses, with an average length of service of 17.62 years (±11.73). Most had a weekly workload of up to 30 hours (70.3%, n=52), worked at night shifts (56.8%, n=42) and did not have another job (54.1%, n=40). In addition, the subjects did physical activity (56.8%, n=42), had leisure time (78.4%, n=58), and showed dissatisfaction with sleep (54%, n=40).

Table 1 presents the means, SD, the risk of illness and the α of the factors of the Work Context Assessment Scale, according to the evaluation of the nursing workers of the psychiatric hospital.

**Table 1:** Risk classification for each factor of the work context of a psychiatric hospital. Teresina, PI, Brazil, 2016

Factor	Mean	SD*	α^†^	Risk
Social-Professional Relationships	2.28	0.820	0.775	Satisfactory
Organization of work	2.62	0.594	0.561	Serious
Working Conditions	3.74	1.073	0.905	Severe

*SD - Standard deviation; †α - Cronbach’s alpha

The organization of work was considered an aspect with serious risk of illness for nursing workers. Social-professional relationships had a satisfactory evaluation. On the other hand, the working conditions received the worst evaluation, presenting severe risk of illness for the nursing workers of the psychiatric hospital. The evaluation of the internal consistency of the ECHT factors demonstrated acceptable values ​​for the factors social-occupational relationships and working conditions. Despite the value lower than 0.70 for organization of work, this factor was kept in the analysis.

Table 2 presents the means and the risk of illness of the EACT items, according to the opinion of the nursing workers of the psychiatric hospital.

**Table 2:** Evaluation of the risk factors for illness related to the work context for nurses in a psychiatric hospital. Teresina, PI, Brazil, 2016

Risk factors for illness			
Item	Mean	SD*	Risk
Organization of Work			
The tasks are repetitive.	4.26	1.135	Severe
The performance is monitored.	3.24	1.515	Serious
The number of people is insufficient to perform the tasks.	3.03	1.647	Serious
There is division between who plans and who executes.	2.82	1.658	Serious
The tasks are performed under deadline pressure.	2.68	1.689	Serious
The results expected are disconnected from reality.	2.34	1.296	Serious
Norms for performing tasks are rigid.	2.28	1.350	Satisfactory
There is strong demand for results.	2.26	1.228	Satisfactory
The pace of work is too fast.	2.08	0.99	Satisfactory
The tasks are discontinued.	2.04	1.318	Satisfactory
There is no time to take breaks and rests at work.	1.77	1.129	Satisfactory
Social-professional relationships			
Communication between employees is unsatisfactory.	2.77	1.467	Serious
Employees are excluded from decisions.	2.72	1.531	Serious
The environment lacks integration.	2.57	1.580	Serious
Management doesn’t support my professional development.	2.41	1.404	Serious
Tasks are not clearly defined.	2.26	1.622	Satisfactory
There is competition between employees in the workplace.	2.24	1.441	Satisfactory
There are difficulties in the communication between bosses and subordinates.	2.22	1.388	Satisfactory
Autonomy is non-existent.	2.08	1.352	Satisfactory
The information I need to perform my tasks are difficult to access.	1.81	1.224	Satisfactory
The distribution of tasks is unfair.	1.72	1.188	Satisfactory
Working Conditions			
Working conditions pose risks to the safety of people.	4.27	1.162	Severe
The furniture in the workplace is inadequate.	3.92	1.478	Severe
There is a lot of noise in the work environment.	3.86	1.388	Severe
The workplace is not suitable for the tasks.	3.81	1.477	Severe
The equipment needed to carry out the tasks are precarious.	3.69	1.507	Serious
Working conditions are precarious.	3.64	1.278	Serious
The physical environment is uncomfortable.	3.64	1.652	Serious
The physical space to perform the work is inadequate.	3.58	1.588	Serious
Consumption material is insufficient.	3.53	1.519	Serious
The work instruments are insufficient to carry out the tasks.	3.46	1.510	Serious

*SD - Standard Deviation

In the context of nursing work in the psychiatric hospital, the items referring to the repetitiveness of tasks, the working conditions that pose risks to the workers’ safety, the furniture and physical structure of the inadequate workplace and the existence of noise in the work environment were those who were considered as a severe risk of illness for the worker.

Table 3 presents the correlation matrix between the factors organization of work, social-professional relationships and working conditions.

**Table 3:** Correlation matrix between the nursing working factors in a psychiatric hospital. Teresina, PI, Brazil, 2016

**Factor**	**Organization of Work**	**Social-professional Relationships**	**Working Conditions**
**Organization of Work**	**1.000**		
**Social-professional Relationships**	0.271*	**1.000**	
**Working Conditions**	**- 0.075**	**0.221**	**1.000**

*Significant correlation was at the level of 0.05.

The organization of work presented a low and direct correlation with the socio-professional relationships (r=0.271, p<0.05). This means that the more negative the evaluation of the organization of work, the more negative the evaluation of the socio-professional relationships.

## Discussion

The EACT is composed of three interdependent factors: organization of work, social-professional relationship and working conditions^11^. The discussion of each of the factors will be presented sequentially, considering the risk of illness for the nursing worker of the psychiatric hospital.

The organization of work in the psychiatric institution studied presented a serious risk for the nursing worker’s illness. This result was similar to that found in a study in a hemodialysis service^6^ with health care workers in basic care^9^, which was still considered positive compared with the severe evaluation reported by intensive care nurses^4^. This result is a warning signal, an indicator of a threshold situation, which may increase work suffering and require immediate attitudes for its confrontation.

Among the 11 items related to the organization of work, the one that obtained the highest mean, indicating severe risk of illness, was “the tasks are repetitive,” which was also observed in other studies with nursing workers^6^ ^,^ ^13^ and with healthcare workers^9^ ^,^ ^14^. High task repetition, evaluated as a severe risk of illness in the organization of the nursing work in the psychiatric institution, deserves to be highlighted, since it is pointed out in studies as a trigger for musculoskeletal injuries, fatigue, monotony, anger and tiredness^6^ ^,^ ^15^ ^-^ ^16^.

Research in a psychiatric hospital showed that, among ergonomic risks at workplace, the monotony/repetitiveness of work is one of the factors most reported by workers^17^. This situation may be related to the experience that nursing workers have in the mental health field of nursing, which consists of repetitive and mechanical movements and is full of rules, routines, norms and based on performing tasks without feelings^18^.

Among the eleven items in the work organization factor, five were considered serious risks for worker’s illness: “*the performance is monitored*”, “*the number of people is insufficient to perform the tasks*”, “*there is division between who plans and who executes*”, “*the tasks are performed under deadline pressure*” and “*the results expected are disconnected from reality*”. These results indicate that the Taylorist model may still be very common in nursing work, since these characteristics are examples of the model.

A study with nursing workers in an emergency service in the city of Natal found that the first condition reported by the workers as unsatisfactory was the fragmentation of work, which involves the division of labor, division of tasks, elements of control and exploitation of the structure of capital^19^.

In nursing, the fragmentation of work is intensified when the professional graduation itself determines the division of tasks and the level of control over work. While nurses are responsible for intellectual work, management, supervision, and control of the whole care process, workers without a college degree are responsible for manual labor, with the function of executing tasks delegated^20^.

As mentioned, the insufficient quantity of human resources was considered a serious risk of illness for the nursing worker. A study pointed out deficiency of professionals as one of the obstacles to the consolidation of a mental health policy in the context of psychiatric hospitals, along with other issues related to political and institutional management^21^. Staff-sizing is a responsibility of the nurse; however, this debate is still incipient in the context of mental health, while in other areas it is already advanced^22^.

The need for patient care is at the core of this discussion, since, despite the fact that the patient with mental disorder does not present physical alterations, the psychological changes related to mood, sleep, personality, aggression and unpredictability of actions require a permanent state of alert. In addition, this field is not attractive for the professionals, due to the stigma and prejudice with the patient with mental disorder.

Resolution No. 527, from November 3^rd^, 2016, which revoked Resolution 293/2004, both from the Federal Nursing Council (COFEn)^22^, updated the parameters for nursing staff sizing and included concepts and calculation methodologies for nursing staffing for mental health services^23^.

The item “*the results expected are disconnected from reality*” discussed in this study was considered as serious risk of illness. Studies point out that issues inherent to psychiatry are sources of frustration and suffering for the worker, causing dissatisfaction and dismay about the work performed, since few changes can be made and visualized by these professionals^24^ ^-^ ^25^.

This frustration is evident when the patient is only pathologized and monitored and also when the revolving door phenomenon is constant, which is characterized by frequent readmissions, which indicate failure in the continuity of treatment by the user and by the management of the Psychosocial Care Network (RAPS)^21^.

“*Norms for performing tasks are rigid*”, “*the pace of work is too fast*”, “*the tasks are discontinued*” and “*there is no time to take breaks and rests at work.*” were considered satisfactory items that can favor the health of the worker. This situation is difficult to understand, because these are items that are related to a rigid model of administration that makes the worker exhausted. However, in this study, these items did not represent conditions unfavorable to the health of the worker.

It is evident that the organization of work can have a significant role in worker illness among nurses in mental health. In addition, despite the classifications of serious risk of illness, this study presented positive items when compared to the intensive care sector^4^ and basic health care^9^, which did not demonstrate any item with satisfactory evaluation in this factor.

In the present study, social-professional relationships were evaluated as satisfactory for risk of illness among nursing workers in mental health. Similar results were found with nursing workers in a hemodialysis service^6^ and nursing workers of a mobile emergency service^26^. This satisfactory evaluation diverges from the results of studies conducted with intensive care workers in Rio de Janeiro^4^ and Rio Grande do Norte^13^, and primary health care nurses in Minas Gerais^27^, for whom the social-professional relationships were evaluated as serious risks of illness. In this same direction, a study with workers from primary health care teams in the Federal District evaluated social-professional relationships as severe risk of illness^14^.

In the social-professional relationships factor, none of the 10 items obtained a severe evaluation for risk of illness. The items evaluated as serious for risk of illness were “*employees are excluded from decisions*”, “*the environment lacks integration*”, “*management doesn’t support my professional development*” and “*communication between employees is unsatisfactory*”, which presented higher mean scores.

Phenomenological studies with nursing workers of a psychiatric inpatient unit in Rio Grande do Sul concluded that the relationship with the other can be a factor of exhaustion, felt and manifested by the worker through his body. In the unit studied, the ambiguous relationships, sometimes happy, sometimes conflictual, define mental health care as the encounter with the other^18^ ^,^ ^28^. The lack of integration among nursing staff is also a source of suffering, and conflicts are clear manifestations of power that must be understood whether they are expressed or veiled^29^.

The high means in the items “*communication between employees is unsatisfactory*” and “*the environment lacks integration*” confirm that many problems present in health care come from inappropriate interpersonal relationships, such as poor communication between employees. Lack of communication can result in feelings of worthlessness, depression, irritation, emotional exhaustion, professional devaluation, and work overload^6^.

A study with nursing workers from the Family Health Strategy (FHS) demonstrated that the worse the evaluation of social-professional relationships, the greater the risk of illness, emotional exhaustion and development of insensitive attitudes^27^.

Communication goes beyond coding of a message; it involves body language, touch, writing, visual contact and posture, and it is a work tool for the nursing professional, not restricted to his connection with the patient, but related to all aspects involved in the caring process. Thus, the importance of social support in the work environment is evident^26^.

The items “*employees are excluded from decisions*” and “*management doesn’t support my professional development*” did not present very high averages. These items were evaluated as serious risk for illness and could mean more flexible management mechanisms. However, moderate/serious evaluations also point to an appreciation of the hierarchy and centralization of decisions, as well as to the discrepancy between work done and work delegated, due to the distance from the decision site and the action site^13^ ^-^ ^14^.

A study with nursing professionals from a specialized mental health service for comprehensive care of users of alcohol, crack and other drugs in São Paulo pointed out that managers who share the leadership with the team of workers are more effective and provide professionals with greater job satisfaction and lower perception of physical and psychic workload^5^. Thus, even with its positive evaluation, the social-professional relationships point to a clear separation between who plans and who executes the tasks, a common feature of the Taylorist administration model.

The items “*tasks are not clearly defined*”, “*there is completion between employees in the workplace*”, “*there are difficulties in the communication between bosses and subordinates*”, “*autonomy is non-existent*”, “*the information I need to perform my tasks are difficult to access*” and “*the distribution of tasks is unfair*” were all considered satisfactory by the nursing staff of the institution studied.

Despite the fact that there is a clear division between planning and execution of the tasks, the results showed that the relationship problems among the workers are more expressive in the same hierarchical level when compared to the bosses. This fact was observed by the lower mean for the item “*there are difficulties in the communication between bosses and subordinates*”, while the items referring to communication, integration and competition between employees obtained higher mean, representing a greater risk for worker illness.

In a psychiatric institution in the state of São Paulo, strong control over work, rigidity and hierarchy in the decision process and relative valorization of the workers were observed. According to the authors, organizational cultures that value the control and specialization of activities, with rigid and vertical organization and centralization of the decision process, have environments that do not allow the development of individual skills of the workers. Therefore, workers are exposed to tensions, feelings of dissatisfaction, demotivation, psychic suffering, emotional exhaustion, depersonalization and lack of personal fulfillment, which leads to mental illness^30^ ^-^ ^31^.

Nursing workers assessed the working conditions as a serious risk for illness. Moreover, among the 10 items used to evaluate this factor, none had a satisfactory/positive evaluation. The results of the present study differ from others conducted in private and public institutions^4^ ^,^ ^6^ ^,^ ^9^ ^,^ ^13^ ^-^ ^14^ ^,^ ^32^. In private care institutions for critically ill and hemodialysis patients, the evaluation was satisfactory, while in public institutions of basic and intensive care the evaluation was serious/moderate. These data indicate the public institutions face difficulties to provide adequate environment and material.

The items “*working conditions pose risks to the safety of people*”, “*the furniture in the workplace is inadequate*”, “*there is a lot of noise in the work environment*” and “*the work place is not suitable for the tasks*” presented the highest averages, representing severe risk for worker’s illness.

Similar results were found regarding the risks to the safety of people, inadequate furniture and excessive noise in a study with nursing professionals of an intensive care unit in Natal, Rio Grande do Norte^13^, and a study in a hemodialysis service in Santa Maria, Rio Grande do Sul^6^.

A study conducted in two hospitals located in Egypt considered the physical and psychosocial workload of the nursing team as stressors and possible causes of illness, absenteeism, work shifts and worse physical and psychological health^33^.

The precariousness of working conditions in psychiatric institutions has been observed for some years, a fact that has stimulated the Psychiatric Reform. A qualitative study with nursing workers from a psychiatric inpatient unit in Rio de Janeiro pointed out that, in addition to the psychological burden inherent in professional work in mental health, there are also burdens related to inadequate working conditions such as lack of material resources, structural inadequacies in terms of physical space, qualitatively and quantitatively insufficient equipment and a reduced number of professionals^34^.

A study conducted with 200 health professionals in a General Public Hospital in Greece points out that a notable lack of workplace stress management strategies is perceive as a lack of interest on behalf of the management regarding the emotional state of the health care workers, which can influence healthcare professionals’ physical and emotional well-being, curbing their efficiency and having a negative impact on their overall quality of life^35^. The lack of material and human resources results in feelings of anguish, tension, anxiety, instability and dissatisfaction. In this way, poor working conditions have a negative impact on the quality of life of professionals^19^ ^,^ ^32^.

In a work context where work conditions are inadequate, nursing professionals are exposed to occupational hazards both for themselves and for patients. In addition, precarious conditions lead to work overload, to a feeling of waste of the vocation of the service, and to underutilization of technical preparation^36^.

It is important to consider the item “*there is a lot of noise in the work environment*”.

Continuous exposure to intense noise, with a mean of 85dB (A) for eight hours a day, can cause structural and functional alterations in the inner ear, leading to noise-induced hearing loss (NIHL). For every 5dB(A) increase after the 85dB(A), the exposure time should be reduced by half. Noise can be considered a public health problem, since NIHL decreases the quality of life, either at work, social activities and with family, and also because it can cause extra-auditory effects. Therefore, WHO recommends a hospital noise level of 40dB(A) during the day and 35dB(A) at night^37^.

Besides changes in hearing, noise can cause disturbances in cardiovascular and respiratory functions, sleep patterns, psychological symptoms and illness, acting as a systemic stressor^38^. Because they spend a lot of time in hospitals throughout their careers, nursing professionals are strongly affected by the excessive levels of occupational noise in a work environment that should be healthy and healing^39^.

Studies in an intensive care unit^4^ and in a hemodialysis service^6^ showed high means in the noise item. These results are consistent and acceptable in sectors characterized by closed environments with unfavorable local acoustics, air conditioning and alarm sounds essential for safe patient care, since they facilitate the quick identification of abnormal situations.

It should be noted, however, that even in the absence of conditions such as those previously mentioned, psychiatric services have their particularity: the type of patient. It is known that the patient with mental disorder can present symptoms that are expressed by elevation of the voice, logorrhea, tachyphrasia, loquacity, glossolalia, screaming, crying and teeth grinding.

The influence of work organization in health reveals that contexts with worse working conditions, failed organization and bad social-professional relationships are associated with physical, emotional and social problems. Among nursing workers, a study pointed out that such factors are related to work overload, conflicts in the work environment, ambiguity in the execution of tasks, lack of professional recognition and aggression^40^.

Caring for patients with mental disorders exposes professionals to aggressions, which may be behaviors or actions with the potential to harm or injure others, either physically or verbally. These aggressions favor the development of anxiety, fear, guilt, sleep disturbances, burnout, low self-perceived health status, work dissatisfaction and stress, with consequent reduction in life satisfaction^41^ ^-^ ^42^.

A study conducted in Israel with 230 mental health nurses, revealed that almost all experienced verbal violence and, more than half, experienced physical violence perpetrated by the patients^42^. A study conducted in Finland pointed out that the prevalence of nurses who experience patient aggressions is higher among professionals working in mental health settings than among those in medical or surgical settings. Working in psychiatry also includes greater odds for diagnosed depression, antidepressant medication use and sick leave due to depression and mental disorders^41^.

The correlated findings demonstrate the importance of work organization, which becomes evident when there is a recurrence of conditions related to demand, pace and pressure, denoting the clear influence of the Taylorist administration model. Studies also identified that nurses perceived harmful levels of psychosocial factors in the work environment, among which were lack of defined roles, lack of autonomy and little social and instrumental support from colleagues and superiors^4^ ^,^ ^43^.

In mental health care, these factors are exacerbated by inflexible and often unquestionable norms, inherited from traditional models of care, with subjective work issues left in the background^18^ ^,^ ^28^. Thus, organizational aspects are more relevant for the development of work-related illnesses than individual factors^4^.

Based on the findings of this study, we recommended that educational institutions provide nursing students with a Workers Health class, with a focus on the issues inherent to the work context and its implications for the health of the worker. In addition, managers of hospital institutions should recognize that professionals exposed to some characteristics of the work environment, such as the repetitiveness of tasks, inadequate furniture, noise and inadequacy physical structure are more vulnerable to work-related illnesses. In addition, nursing workers should take part, as co-responsible for their health, in events, meetings, and research related to workers’ health, and the organizations of this class should supervise the institutions regarding compliance with safety standards, providing assistance to workers, reinforcing the importance of caring for oneself and encouraging research on workers’ health.

The limitations of this study are related to its design, which does not allow defining cause and effect, its small sample size, which precludes a more detailed statistical analysis, and the scarcity of studies with the population of nursing workers of psychiatric hospitals, a limitation that was minimized by the use of research with nursing professionals from other settings.

## Conclusion

The analysis allowed observing that the factor working conditions is the one that most contributes to illness among nursing workers. In addition, the organization of work showed a predominance of the Taylorist administration model, which contributes moderately to illness, but is a warning signal for situations that can lead to work suffering. It should be noted, however, that the social-professional relationships factor was significantly positive.
